# Pharmacogenomic impact and genetic architecture of toxicity in pediatric acute lymphoblastic leukemia induction therapy: an exploratory modeling approach

**DOI:** 10.3389/fphar.2026.1830183

**Published:** 2026-06-16

**Authors:** Carolina Gutierrez-Cáceres, Claudio Alarcón-Concha, Javiera Aracena-Farías, Esperanza Torres, Iván Acuña, Tamara Sandoval, Pamela Silva, Maricruz Ormeño, Verónica Oyarce, Leslie C. Cerpa, Matías F. Martínez

**Affiliations:** 1 Laboratory of Advanced Research in Personalized Medicine, Department of Pharmaceutical Sciences and Technology, Faculty of Chemical and Pharmaceutical Sciences, University of Chile, Santiago, Chile; 2 Faculty of Pharmacy, University of Concepcion, Concepcion, Chile; 3 Laboratory of Chemical Carcinogenesis and Pharmacogenetics (CQF), Department of Basic and Clinical Oncology, Faculty of Medicine, University of Chile, Santiago, Chile; 4 Hospital de Niños Roberto del Río, Santiago, Chile; 5 Hospital Luis Calvo Mackenna, Santiago, Chile; 6 Hospital Exequiel González Cortés, Santiago, Chile; 7 Centro de Investigación Clínica Avanzada (CICA) Hospital Exequiel Gonzalez Cortés - Facultad de Medicina Universidad de Chile, Santiago, Chile; 8 Center for Cancer Prevention and Control (CECAN), Santiago, Chile; 9 Latin American Network for Implementation and Validation of Clinical Pharmacogenomics Guidelines (RELIVAF-CYTED), Santiago, Chile

**Keywords:** acute lymphoblastic leukemia, adverse drug reaction, pediatric oncohematology, pharmacogenomics, polygenic risk score (PGRS), precision medicine

## Abstract

Induction chemotherapy for pediatric Acute Lymphoblastic Leukemia (ALL) is highly curative but accompanied by a severe toxicity profile. Evaluating the genetic susceptibility to these adverse events could inform hypothesis-generating risk stratification. In this exploratory retrospective cohort study (N = 178), we investigated the influence of 14 candidate pharmacogenetic variants on the development of prevalent Adverse Drug Reactions (ADRs, ≥ 10 events). Multiple logistic regression, adjusted for basic demographic and anthropometric covariates (age and body surface area), was utilized across comprehensive inheritance architectures (additive, dominant, recessive, and co-dominant). For clinical translation hypothesis generation, we applied LASSO-penalized logistic regression (α = 1), 10-fold cross-validation to derive parsimonious Pharmacogenetic Risk Scores (PGRS). Model performance was evaluated using the optimism-corrected Area Under the Receiver Operating Characteristic Curve (AUC) via 1000-iteration bootstrap resampling, and calibration was assessed via the Brier score. We observed several complex interactions, notably suggesting an increased risk of severe neutropenia in patients carrying a homozygous variant *CYP3A5* rs776746 genotype (nominally significant OR 5.58, p = 0.006) and a potential pleiotropic protective effect from *NFATC2* rs6021191 (OR 0.09, p = 0.001). Penalized selection allowed the construction of compact, multi-locus mathematical risk scores that demonstrated promising diagnostic discrimination for Neutropenia (optimism-corrected AUC 0.793, 95% CI 0.72–0.85; Brier score 0.12), while displaying limited to moderate performance for other clinically discriminating toxicities like Infection (AUC 0.650) and Gastrointestinal complications (AUC 0.607). The primary contribution of this study is the prioritization of pharmacogenomic candidate signals rather than the proposal of models ready for clinical intervention. The integration of alternative genotype parameterizations and regularized machine learning highlights pathways for future predictive research. However, given the retrospective nature of the study and inherent risks of internal optimism, these scores serve as biologically informed proof-of-concept models that strictly require robust prospective external validation.

## 1 Introduction

Acute lymphoblastic leukemia (ALL) is the most common malignancy in childhood. In high-income countries, contemporary risk-adapted therapy has raised survival above 90%; however, this progress has not been equitably reproduced across all populations. In Latin American and other low- and middle-income settings, outcomes remain more heterogeneous and, in many centers, substantially poorer, with reported survival ranges far below those observed in high-income countries. In parallel, Hispanic children bear a disproportionate burden of ALL incidence and have repeatedly been described as a population with less favorable outcomes, reflecting a combination of biological, socioeconomic, and health-system factors. This persistent disparity reinforces the need for translational strategies responsive to the genetic architecture, treatment context, and implementation realities of underrepresented regional populations (Duffy et al., 2023; Rivera-Luna et al., 2022).

Although Chile-specific population-level estimates for pediatric ALL and treatment-related mortality are limited in international cancer databases, available national and regional data provide relevant epidemiological context. According to GLOBOCAN 2022, leukemia accounted for an estimated 1,432 new cases and 879 deaths in Chile, ranking 13th in cancer incidence and 10th in cancer mortality among all cancer sites; these estimates include leukemia across all ages and are not specific to pediatric ALL. At the pediatric ALL level, the clinical importance of toxicity prediction is more directly supported by regional outcome studies. In a recent multicenter South American pediatric ALL cohort, induction death occurred in 6.4% of patients (48/746), with infection accounting for 89.6% of induction deaths (43 cases); remission death occurred in 3.8% (29/746), and among remission deaths occurring while patients were still on therapy, 80% (16/20) were attributed to toxicity. These data highlight that predicting severe treatment-related toxicity in pediatric ALL has potential clinical relevance not only for supportive care optimization, but also for reducing preventable treatment-related mortality in regional settings (Ferlay et al., 2024; Duffy et al., 2023).

The remission induction phase—commonly structured with a backbone of glucocorticoids, vinca alkaloids, anthracyclines, and L-asparaginase—frequently triggers systemic morbidity, profound bone marrow aplasia, and severe infectious complications (Inaba et al., 2013; Pui and Evans, 2006). Despite major therapeutic advances, clinically meaningful interindividual variability in induction-related toxicity persists and remains a key contributor to treatment interruptions, dose modifications, and short- and long-term morbidity.

Interindividual variability in the incidence of Adverse Drug Reactions (ADRs) strongly suggests an underlying genetic susceptibility. While pharmacogenomic approaches have successfully optimized the maintenance phases of ALL treatment—most notably reflected in the Clinical Pharmacogenetics Implementation Consortium (CPIC) guidelines for *TPMT* and *NUDT15* genotyping to guide thiopurine dosing (Adam de Beaumais et al., 2011; Relling et al., 2019; Maillard et al., 2026)—the toxicity risk associated with multi-agent induction regimens remains less firmly established in routine clinical practice (Zgheib et al., 2013). Importantly, the established clinical actionability of TPMT and NUDT15 is primarily linked to thiopurine exposure, particularly mercaptopurine, thioguanine, and azathioprine. Therefore, while these genes represent high-evidence pharmacogenetic markers in pediatric ALL therapy, their direct relevance is phase- and drug-dependent. This distinction is particularly important for induction-focused analyses, where the main toxicity profile is shaped by a multi-agent backbone that differs from thiopurine-intensive maintenance therapy.

Biotransformation enzymes, such as the Cytochrome P450 family (CYP3A5), are directly implicated in the clearance of vincristine and dexamethasone (Hossam Abdelmonem et al., 2024). Furthermore, membrane transporters (SLC28A2, ABCB1, OAT4) and modulators of innate immunity and inflammation (TLR2, NFATC2, IL6) play critical roles in tissue-specific pharmacokinetics and the microenvironmental response to cytotoxic stress (Chen et al., 2024; Germain et al., 2022; Kotlyarov and Kotlyarova, 2022; Martinez et al., 2020). Importantly, the pharmacogenomic associations across populations may be limited by differences in allele frequencies, underscoring the need for evidence generation in underrepresented groups such as Latin American pediatric cohorts.

The primary objective of this exploratory study is to perform a candidate-variant pharmacogenetic profiling of toxicity during ALL induction in a Chilean pediatric cohort, while contextualizing the findings within the broader need for pharmacogenomic evidence generation in underrepresented Latin American settings, controlling for basic demographic and anthropometric covariates. Recognizing the common analytical challenges of genetic collinearity, multiple testing, and small-sample bias in rare events, we restricted our main analysis to prevalent toxicities. Furthermore, we utilized regularized Machine Learning methodologies (LASSO) to construct parsimonious Pharmacogenetic Risk Scores (PGRS) intended to prioritize variables for future predictive clinical modeling and prospective validation studies (Steyerberg et al., 2010).

## 2 Materials and methods

### 2.1 Study design and patient population

This was an analytical observational study based on a retrospective cohort utilizing consolidated clinical records from the *U-INICIA* project (Universidad de Chile). The inclusion criteria required pediatric patients (<18 years old) diagnosed with *de novo* ALL who received standardized remission induction chemotherapy based on the IA and IA* protocols (Intercontinental ‘BFM'-based backbone) between March 2022 and December 2023. A total of 178 patients met the inclusion criteria and had matching clinical and genotypic records. The study was conducted in accordance with the Declaration of Helsinki, and the protocol was approved by the local Institutional Review Board of the Faculty of Medicine of the University of Chile (9 November 2021; project 103-2021; act n°099). Informed consent was obtained from parents or legal guardians, and informed assent was obtained in children older than 10 years.

### 2.2 Clinical variables and phenotype definitions

Data extraction included baseline demographics, anthropometrics, and disease characteristics. To adjust for baseline characteristics, Age at diagnosis and Body Surface Area (BSA) were included as mandatory clinical covariates in all models. Missing values in these continuous covariates (< 5% overall) were imputed using the sample mean.

Initial clinical risk stratification was abstracted from the treating-center medical records and reported as a baseline cohort descriptor. However, because the present study focused specifically on remission induction, patients received a common IA/IA* Intercontinental BFM-based induction backbone before definitive post-induction risk reclassification and subsequent treatment-intensity adaptation. Therefore, risk group was not modeled as a proxy for induction-dose intensity in the primary analyses. Incorporating post-induction or response-informed risk assignment into models of induction toxicity could introduce inappropriate post-baseline adjustment.

Seventeen ADR domains were abstracted from the medical records and graded according to CTCAE version 5.0. For each ADR, the highest grade observed during the remission induction phase was retained for analysis. Laboratory-based toxicities (e.g., anemia, neutropenia, thrombocytopenia, electrolyte abnormalities, and hepatobiliary abnormalities) were classified using CTCAE laboratory thresholds. Clinically defined toxicities (e.g., infection, gastrointestinal toxicity, L-asparaginase hypersensitivity, thrombosis, and neurotoxicity) were identified from physician-documented diagnoses and graded according to CTCAE-compatible clinical criteria whenever applicable. For statistical purposes, ADRs were dichotomized as low/moderate toxicity (CTCAE grades 0–2) versus severe/critical toxicity (CTCAE grades 3–5) according to their occurrence during the induction phase. ADR domains were coded independently rather than hierarchically; therefore, overlapping events were allowed, such that, for example, infection occurring in a patient with neutropenia could contribute to both outcomes if each met the grade ≥3 definition (Supplementary Table S1).

To prevent small-sample bias, complete separation (infinite maximum likelihood estimates), and severe overfitting, rare toxicities with a population prevalence of < 10 events (e.g., Anaphylaxis, Neurotoxicity, Hepatobiliary, Thrombosis) were excluded from the main predictive modeling and relegated to the Supplementary Material (evaluated via Firth’s penalized logistic regression to account for quasi-complete separation) (Firth, 1993). The main manuscript focuses exclusively on prevalent ADRs (≥10 events).

### 2.3 Genotyping, quality control, and inheritance modeling

Genomic DNA was extracted, and 14 candidate genetic variants were genotyped using TaqMan® allelic discrimination assays (ThermoFisher). Genotyping quality control included marker-specific assessment of successful genotypic calls and Hardy–Weinberg equilibrium (HWE) testing for each locus with available genotype data. Marker-level HWE p-values are reported in Table 3. Because some loci showed sparse genotype categories, absent rare homozygotes, or low valid call counts, HWE results were interpreted cautiously as quality-control descriptors rather than as definitive evidence of genotyping failure.

The 14 variants evaluated in this study were selected using a tiered candidate-gene strategy. First, we included pharmacogenes with established clinical relevance in ALL-related thiopurine toxicity, particularly TPMT and NUDT15, which have guideline-supported clinical actionability and remain the clearest example of successful pharmacogenetic implementation in pediatric ALL. Second, because validated pharmacogenomic markers for induction-phase multi-agent toxicity remain limited, we incorporated additional loci based on prior candidate-gene literature and biological plausibility related to drug metabolism, transport, inflammation, and host-response pathways potentially involved in severe treatment-related toxicity during induction. Accordingly, the present work should be interpreted as an exploratory signal-prioritization study rather than as the validation of a fully guideline-qualified biomarker panel for immediate clinical deployment (Maillard et al., 2026; Relling et al., 2019; Hossam Abdelmonem et al., 2024; Chen et al., 2024).

To evaluate alternative statistical encodings of genotype effect, each variant was parameterized under additive, dominant, recessive, and co-dominant models. In this study, these terms refer strictly to statistical genotype parameterizations and should not be interpreted as definitive biological inheritance mechanisms. The co-dominant parameterization was used to estimate heterozygous and homozygous variant effects separately relative to the reference genotype. These models were evaluated in parallel for exploratory signal prioritization rather than to infer a single causal mode of inheritance for each variant.

The candidate-variant panel included loci with heterogeneous types of biological support: some variants were selected because of established or plausible roles in drug disposition (e.g., CYP3A5), whereas others were prioritized because of their potential involvement in immune and inflammatory response pathways relevant to treatment-related toxicity (e.g., NFATC2 and TLR2). Consequently, the mechanistic interpretation of any given variant–ADR association was framed according to the known functional category of the variant rather than assuming a direct drug-specific causal pathway for all loci (Krediet et al., 2007; Fernandez et al., 2015; Bains et al., 2013). Although TPMT and NUDT15 were included because of their established clinical relevance for thiopurine-associated toxicity, they were analyzed as candidate pharmacogenetic variants rather than as mandatory adjustment covariates. This decision was based on the induction-specific focus of the study, since the strongest evidence for TPMT/NUDT15-guided prescribing relates to thiopurine exposure, particularly 6-mercaptopurine, which is not the principal systemic drug exposure during the induction window analyzed here.

### 2.4 Statistical analysis

The analytical pipeline consisted of two distinct phases.

1. Adjusted Parametric Exploratory Models: Standard multivariable logistic regression was performed for Variant-ADR interactions, continuously controlling for Age and BSA. Odds Ratios (OR) and unadjusted nominal *p*-values (p < 0.05) are reported. Due to the exploratory, hypothesis-generating nature of this study, we report unadjusted *p*-values while acknowledging the risk of false positives inherent to multiple comparisons. Candidate pharmacogenetic variants were not used as fixed adjustment covariates for one another in the primary single-variant models. Instead, they were evaluated as predictors under a common exploratory framework, and multivariable penalized modeling was used to assess whether combinations of variants contributed to parsimonious PGRS construction.
2. Parsimonious PGRS Construction and Internal Validation: To construct multi-locus scores while actively mitigating overfitting, the variant space was pre-filtered using a soft univariate threshold (p < 0.25). The resulting design matrix was standardized and subjected to LASSO-penalized logistic regression (Least Absolute Shrinkage and Selection Operator, α = 1). The optimal penalization hyperparameter (λ) was selected using 10-fold cross-validation, employing the most restrictive penalty within one standard error of the minimum (lambda.1se rule) to enforce strict sparsity (Friedman, et al., 2010). The non-zero coefficients extracted were then refitted in an unpenalized logistic model to derive the final weights (ln (OR)).

To provide a robust estimate of internal validity and account for potential model overfitting, an optimism-corrected AUC and its 95% Confidence Intervals (95% CI) were calculated using a rigorous 1000-iteration bootstrap resampling procedure. Calibration was assessed using the Brier score (ranging from 0 to 1, where lower values indicate better calibration and prediction accuracy). The Youden Index was applied to extract the theoretical optimal Sensitivity and Specificity thresholds for the current dataset. The study is reported in accordance with TRIPOD (Collins et al., 2015) using R version 4.3 (packages glmnet, pROC, rms).

Given the exploratory aim and the limited number of events for several ADRs, primary models used a parsimonious covariate structure adjusted for age at diagnosis and BSA. Additional clinical variables, including sex, leukocyte count, cytogenetic/molecular subtype, treatment-intensity modifiers, and therapeutic risk stratification, were considered clinically relevant but were not forced into the primary models to preserve model stability. These variables are acknowledged as sources of residual confounding and are prioritized for future clinical–pharmacogenomic prediction models. Risk stratification was not included as a mandatory adjustment covariate because, within the induction window analyzed, treatment intensity was protocol-based and broadly common across patients, whereas definitive risk-adapted intensification occurs after induction response assessment.

Accordingly, nominal statistical significance was used for signal prioritization in this hypothesis-generating framework and not as the sole criterion to define a uniquely optimal inheritance structure. Future confirmatory studies should compare alternative genetic models using formal fit and parsimony criteria, such as AIC or BIC, together with biological plausibility and external reproducibility.

The overall study design and analytical workflow are summarized in Supplementary Figure S1.

## 3 Results

### 3.1 Baseline demographics and prevalent ADRs

The final cohort comprised 178 pediatric patients with a mean age at diagnosis of 5.4 years. The distribution of sex, initial risk stratification, and baseline clinical parameters are summarized in Table 1.

**TABLE 1:** Baseline clinical and sociodemographic characteristics of the study cohort.

Characteristic	n = 178 (100%)
Age at diagnosis (years), mean (±SD)	5.40 (± 3.42)
Body surface area (m^2^), mean (±SD)	0.67 (± 0.24)
Sex (male/female)	96 (53.9%)/ 82 (46.1%)
Initial risk stratification	
Standard risk	63 (35.4%)
Intermediate	101 (56.7%)
High risk	7 (3.9%)
Unknown/ Missing	7 (3.9%)
Central nervous system (CNS) status	
CNS 1 (no blasts)	140 (78.7%)
CNS 2 or 3 (involvement)	27 (15.2%)
Unknown/ Missing	11 (6.2%)

As shown in Table 1, the cohort was composed of 178 pediatric patients, with a balanced sex distribution and a predominance of standard and intermediate risk categories. High-risk patients represented a small subgroup, which was considered in the interpretation of risk-stratified clinical heterogeneity.

Induction toxicity was largely dominated by bone marrow failure and systemic immunosuppression. Following our methodological criteria, rare ADRs (<10 events) were relegated to Supplementary Table S2. The prevalent toxicities analyzed in the main study are detailed in Table 2.

**TABLE 2:** Prevalence of main Adverse Drug Reactions (ADRs, ≥10 events).

Adverse drug reaction (ADR)	Events, n (%)
Anemia	169 (94.9)
Neutropenia	165 (92.7)
Thrombocytopenia	141 (79.2)
Infection	58 (32.6)
Gastrointestinal toxicity	31 (17.4)
L-asparaginase hypersensitivity	26 (14.6)
Electrolyte disorder	11 (6.2)

Table 2 shows that severe hematologic toxicities were highly prevalent during induction, particularly anemia, neutropenia, and thrombocytopenia. In contrast, infection, gastrointestinal toxicity, L-asparaginase hypersensitivity, and electrolyte disorders occurred less frequently and therefore provided more clinically discriminating endpoints for exploratory prediction.

### 3.2 Allelic frequencies and genetic variability

Table 3 summarizes genotype distributions, allele frequencies, and marker-level HWE results for the analyzed variants. The number of valid genotype calls ranged from 17 to 177 across loci, with particularly low genotyping success for CYP3A5 (rs4646450), consistent with technical limitations during probe-based genotyping. Most loci were broadly consistent with HWE; however, CYP3A5 (rs776746), TLR2 (rs4696480), and TPMT (rs1142345) showed deviations from equilibrium and should therefore be interpreted cautiously. In addition, several loci displayed sparse genotype categories or absent rare homozygotes, including NUDT15 (rs116855232), TPMT (rs1800460), and NFATC2 (rs6021191), which limits the informativeness of HWE-based assessment and the stability of some inheritance-specific estimates. Thus, Table 3 provides both the allele-frequency context and the quality-control framework required to interpret the subsequent variant–ADR association analyses.

**TABLE 3:** Comprehensive genotypic and allelic frequencies.

Gene (Genetic variant)	N Valid*	Ref/Ref n (%)	Ref/Var n (%)	Var/Var n (%)	Ref allele freq	Var allele freq	HWE p-value
ABCB1 (rs1045642)	176	CC: 20 (11.4%)	CT: 89 (50.6%)	TT: 67 (38.1%)	C: 0.366	T: 0.634	0.24
CYP3A5 (rs776746)	177	AA: 11 (6.2%)	AG: 32 (18.1%)	GG: 134 (75.7%)	A: 0.153	G: 0.847	<0.05
IL6 (rs1800796)	142	CC: 14 (9.9%)	CG: 59 (41.5%)	GG: 69 (48.6%)	C: 0.306	G: 0.694	0.79
MTHFR (rs181133)	144	AA: 52 (36.1%)	AC: 67 (46.5%)	CC: 25 (17.4%)	A: 0.594	C: 0.406	0.67
CYP3A5 (rs4646450)	17	CC: 1 (5.9%)	CT: 8 (47.1%)	TT: 8 (47.1%)	C: 0.294	T: 0.706	0.58
MTHFR (rs181131)	145	TT: 4 (2.8%)	TG: 52 (35.9%)	GG: 89 (61.4%)	T: 0.207	G: 0.793	0.26
NUDT15 (rs116855232)	174	CC: 161 (92.5%)	CT: 13 (7.5%)	TT: 0 (0.0%)	C: 0.963	T: 0.037	0.61
OAT4 (rs11231809)	135	AA: 9 (6.7%)	AG: 57 (42.2%)	GG: 69 (51.1%)	A: 0.278	G: 0.722	0.54
OATP1B1 (rs11045879)	173	TT: 5 (2.9%)	TC: 37 (21.4%)	CC: 131 (75.7%)	T: 0.136	C: 0.864	0.24
SLC28A2 (rs11854484)	120	CC: 61 (50.8%)	CT: 40 (33.3%)	TT: 19 (15.8%)	C: 0.675	T: 0.325	0.80
TLR2 (rs4696480)	127	TT: 49 (38.6%)	TA: 78 (61.4%)	AA: 0 (0.0%)	T: 0.693	A: 0.307	<0.05
TPMT (rs1142345)	160	AA: 3 (1.9%)	AG: 12 (7.5%)	GG: 145 (90.6%)	A: 0.056	G: 0.944	<0.05
TPMT (rs1800460)	167	GG: 0 (0.0%)	GA: 13 (7.8%)	AA: 154 (92.2%)	G: 0.039	A: 0.961	0.60
NFATC2 (rs6021191)	175	AA: 151 (86.3%)	AG: 24 (13.7%)	GG: 0 (0.0%)	A: 0.931	G: 0.069	0.33

Footnote: N Valid indicates the number of patients with successful genotypic amplification for the corresponding locus. HWE: Hardy–Weinberg equilibrium. HWE p-values should be interpreted cautiously for loci with sparse genotype categories, absent rare homozygotes, or low valid call counts.

Of note, CYP3A5 (rs4646450) showed very low genotyping yield (N valid = 17), which substantially limits the stability and interpretability of inheritance-specific estimates for this locus. Accordingly, this variant was retained for descriptive completeness but was not given inferential emphasis in the interpretation of the main findings.

### 3.3 Exploratory multivariate associations

**FIGURE 1:**
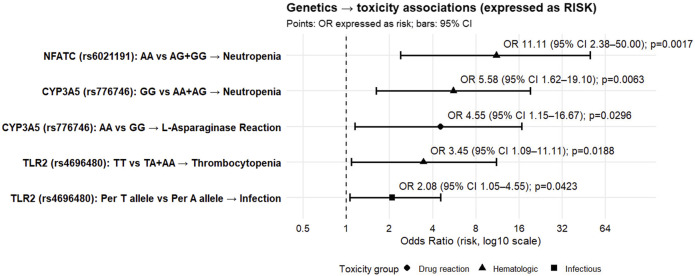
Forest plot of nominally significant associations between genetic variants and prevalent adverse drug reactions during induction therapy in pediatric ALL. The figure displays odds ratios (ORs) and 95% confidence intervals from adjusted multivariable logistic regression models for VARIANT–ADR associations with nominal significance (p < 0.05). Models were adjusted for age at diagnosis and body surface area, and the association shown for each variant corresponds to the inheritance model under which significance was observed (additive, dominant, recessive, or co-dominant). The vertical reference line indicates the null effect (OR = 1). Estimates to the right of the line indicate increased odds of grade ≥3 toxicity, whereas estimates to the left indicate decreased odds. Only ADRs with at least 10 events in the cohort were considered in the main analysis. For visualization purposes, inverse/protective associations were re-expressed as reciprocal ORs so that all estimates are displayed in the direction of increased risk. Original model estimates are provided in Supplementary Table S3.

Statistical control for age, BSA, and inheritance structure unveiled several nominally significant pharmacogenomic associations. Figure 1 summarizes the nominally significant variant–ADR associations observed in the adjusted exploratory models. The adjusted multivariable models underlying the nominally significant variant–ADR associations are detailed in Supplementary Table S3, which reports the effect estimates for the prioritized genetic variants together with age and body surface area.

### 3.4 PGRS construction via penalized machine learning and internal validation

To manage collinearity and enforce parsimony, LASSO regularization (10-fold CV) was applied to the pre-filtered genetic variables alongside clinical covariates. Table 4 summarizes the PGRS formulas and internal performance metrics. The strongest discrimination was observed for neutropenia, followed by anemia, whereas infection, thrombocytopenia, and gastrointestinal toxicity showed more modest performance. These differences likely reflect both endpoint prevalence and the limited clinical covariate structure of the exploratory models.

**TABLE 4:** LASSO-derived PGRS formulas and internal performance metrics.

Target ADR	Parsimonious score formula (extracted weights)	Optimism-corrected AUC (95% CI)	Brier score	Sensitivity	Specificity
Neutropenia	+1.33 * CYP3A5 (GG vs. AG/AA) −1.58 * NFATC2 (per G allele) −1.58 * MTHFR(131) (GG vs. TG/TT)	0.79 (0.72 – 0.85)	0.12	78.8%	69.2%
Anemia	+1.55 * CYP3A5 (GG vs. AG/AA) +1.10 * NFATC2 (per G allele) +1.05 * SLC28A2 (per T allele) −0.72 * OAT4 (per G allele)	0.77 (0.70 – 0.84)	0.10	84.0%	66.7%
L-asparaginase hypersensitivity	−0.69 * CYP3A5 (per G allele) +0.88 * NFATC2 (per G allele) +0.80 * IL6 (GG vs. GC/CC) +0.66 * OATP1B1 (per C allele)	0.69 (0.58 – 0.78)	0.11	80.8%	48.7%
Infection	−0.68 * TLR2 (per A allele) −0.70 * OAT4 (AG/GG vs. AA) −0.35 * IL6 (per G allele)	0.65 (0.56 – 0.73)	0.21	74.1%	50.8%
Thrombocytopenia	−1.32 * TLR2 (TA/AA vs. TT) −0.93 * CYP3A5 (rs4646450) (per T allele) −0.36 * TPMT(rs1142345) (per G allele)	0.63 (0.55 – 0.70)	0.18	32.6%	89.2%
Gastrointestinal	+0.99 * TLR2 (per A allele) +0.63 * ABCB1 (TT vs. TC/CC) −1.26 * OAT4 (GG/AG vs. AA)	0.61 (0.51 – 0.70)	0.15	100.0%	15.6%

Sensitivity and Specificity are reported at the optimal Youden Index threshold. Brier scores closer to 0 indicate better calibration and probabilistic accuracy.

## 4 Discussion

The overarching contribution of this study is the prioritization of pharmacogenomic candidate signals rather than the proposal of a model ready for clinical intervention. The translation of pharmacogenomics into modern hemato-oncology requires evolving from isolated single-mutation analyses to multivariate predictive frameworks. Our exploratory study illustrates that evaluating multiple genotype parameterizations may help identify candidate associations that could be missed under a single assumed genetic model. However, these parameterizations should be interpreted as statistical representations of genotype effect rather than as evidence of a definitive biological inheritance mechanism (Diouf et al., 2015). By utilizing LASSO-penalized regression, we identified parsimonious modules that could inform future PGRS and streamline candidate selection for larger cohorts.

Importantly, the variants prioritized in this study should be interpreted as candidate predictive markers rather than as proven causative determinants of toxicity. For loci such as CYP3A5 rs776746, NFATC2 rs6021191, and TLR2 rs4696480, the observed associations may reflect differences in drug disposition, immune regulation, or host inflammatory response, but the present retrospective exploratory design cannot establish direct causality. Accordingly, these signals are best viewed as risk indicators to be tested in future integrated clinico-genomic models.

In single-variant analyses, nominal associations highlighted CYP3A5 rs776746 and immune-related variants (NFATC2, TLR2) as potential contributors to hematologic and infectious toxicities, while penalized models yielded compact PGRS with the strongest discrimination for neutropenia. Given the exploratory nature of the study, we interpreted these signals through a concordance framework, distinguishing findings that align with established gene–phenotype relationships from those that are biologically plausible but discordant with the most commonly reported toxicity phenotypes.

### 4.1 Clinical utility and the challenge of universal events

It is crucial to contextualize the performance of these models based on the baseline prevalence of the ADRs. During induction, hematological toxicities such as anemia (94.9%) and neutropenia (92.7%) were highly prevalent. Consequently, the practical clinical value of predicting the mere presence or absence of these events is inherently limited. Although the PGRS for Neutropenia exhibited promising discrimination (AUC 0.793), this score should strictly not be interpreted as a standalone triage tool for clinical intervention. Instead, such models serve primarily as *biologically informed proof-of-concept models*. They may help anticipate the intensity, prolonged duration, or heightened need for clinical surveillance, prioritizing these signals for future research into risk-adapted supportive care.

From a translational perspective, the clinical value of toxicity prediction in pediatric ALL should be interpreted in relation to therapy-related morbidity and mortality rather than solely to statistical discrimination. This is particularly relevant during induction, when severe infections and treatment-related complications remain major contributors to early mortality in regional pediatric ALL cohorts (Duffy et al., 2023). Therefore, although the present PGRS should not be used as a standalone clinical decision tool, the prioritization of pharmacogenomic signals associated with severe toxicity may support future integrated clinico-genomic models aimed at identifying patients who require intensified monitoring, early supportive-care interventions, or closer management of treatment complications.

Conversely, for more clinically discriminating toxicities that are less frequent—such as Infection (AUC 0.650) and Gastrointestinal complications (AUC 0.607)—the models demonstrated only limited to moderate performance. This underscores that while the LASSO approach effectively prioritizes variables, these specific scores are meant to highlight pharmacogenetic signals rather than support direct clinical decision-making. Future studies should therefore prioritize richer phenotypes (duration/time-to-recovery, nadir counts, infection-related endpoints) to translate pharmacogenetic signals into decision-relevant predictions.

The interpretation of TPMT and NUDT15 findings also requires phase-specific pharmacological context. These genes have strong clinical evidence for thiopurine-related toxicity and are central to mercaptopurine dose individualization in pediatric ALL. However, the present study focused on remission induction, a treatment window in which severe toxicity is mainly driven by a multi-agent induction backbone rather than by sustained 6-mercaptopurine exposure. Therefore, TPMT and NUDT15 should not be interpreted here as negative controls of established thiopurine pharmacogenetics, nor should the absence of a dominant induction-phase signal be viewed as contradicting their validated role in thiopurine-containing phases. Instead, their inclusion provides continuity with established ALL pharmacogenetics while highlighting the need to identify additional markers relevant to induction-specific toxicity.

### 4.2 The hepatic pathway (CYP3A5)

CYP3A5 represents a biologically relevant gene for induction-phase pharmacology because vincristine is metabolized by CYP3A enzymes, and prior studies have linked low CYP3A5 expression genotypes with increased vincristine-induced neurotoxicity. This aspect is concordant with the established pharmacological role of CYP3A5 in vincristine disposition. However, our main signal involved severe neutropenia rather than neurotoxicity, which is discordant with the phenotype most consistently reported in the literature. Therefore, the CYP3A5–neutropenia association should not be interpreted as a direct mechanistically established effect. Rather, it may reflect indirect consequences of multi-agent exposure during induction, regimen-level interactions, or residual clinical confounding. Similarly, any CYP3A5 signal involving gastrointestinal toxicity should be considered exploratory and indirectly plausible rather than a validated drug–gene–toxicity relationship. Overall, CYP3A5 rs776746 is best interpreted here as a candidate predictive marker of altered vulnerability during induction, not as a direct causal trigger of hematologic or gastrointestinal toxicity (Ahmed et al., 2026; Egbelakin et al., 2011).

Interestingly, the same variant displayed an inverse pattern for L-asparaginase hypersensitivity, where AA (vs. GG) was associated with higher risk (OR 4.55), suggesting comparatively lower risk among GG carriers in this contrast. A biologically plausible explanation is that interindividual variability in the immunosuppressive milieu during induction—potentially influenced by corticosteroid exposure—may modulate adaptive immune priming against asparaginase; however, steroid exposure was not measured in this study and this mechanism warrants prospective evaluation. Overall, this association should be interpreted cautiously, as L-asparaginase hypersensitivity is also shaped by established immunogenetic determinants, most notably HLA haplotypes (e.g., HLA-DRB1), as well as clinical factors such as treatment timing and supportive care practices (Fernandez et al., 2014; Kutszegi et al., 2017). Thus, CYP3A5 rs776746 should be interpreted here as a candidate predictive marker of altered vulnerability during induction rather than as a direct causal trigger of myelosuppression.

### 4.3 Innate immunity and cellular resilience

Immune-regulatory variants also emerged among the prioritized exploratory signals. These findings should be interpreted primarily as host-response markers rather than as direct pharmacologic causes of toxicity. For NFATC2 rs6021191, the most concordant external evidence relates to asparaginase hypersensitivity. Prior genome-wide analyses in pediatric ALL identified rs6021191 as an intronic regulatory variant associated with higher NFATC2 expression and increased risk of asparaginase hypersensitivity, supporting an immune-regulatory mechanism rather than a structural protein-disrupting effect (Fernandez et al., 2015). In this sense, the relevance of NFATC2 in the present study is most biologically coherent when interpreted within immune activation and treatment-response modulation.

For TLR2, the association with thrombocytopenia is biologically plausible through the role of innate immune signaling in megakaryocyte and platelet biology. TLR2 is expressed in megakaryocytes and platelets, and experimental data indicate that TLR2 stimulation can activate NFκB, ERK-MAPK, and PI3K/Akt pathways in megakaryocytic cells, with downstream effects on megakaryocyte maturation markers (Beaulieu et al., 2011). In addition, inflammation, infection, and cytokine signaling can modulate megakaryopoiesis and platelet production (Couldwell and Machlus, 2019). Therefore, a TLR2-related signal for thrombocytopenia may reflect altered host inflammatory response within the chemotherapy-stressed bone marrow microenvironment. Nevertheless, this interpretation remains indirect and exploratory, and should not be viewed as evidence that TLR2 rs4696480 directly causes chemotherapy-induced thrombocytopenia.

Taken together, these immune-related signals reinforce the potential value of integrating host-response markers into future clinical–pharmacogenomic prediction models. However, the present findings include both concordant signals, such as the NFATC2 relationship with asparaginase hypersensitivity, and more indirect or discordant signals, such as CYP3A5 with hematologic toxicity. This balance supports their use for prioritization and hypothesis generation, but not for causal inference or immediate clinical decision-making.

To support a more rigorous mechanistic interpretation and reduce the risk of overstatement, the prioritized loci are summarized in a supplementary drug–gene–toxicity concordance matrix, which distinguishes established pharmacologic links from indirect immune-regulatory or exploratory biological plausibility (Supplementary Table S4).

### 4.4 Limitations

While our analytical pipeline utilized rigorous L1 regularization and internal cross-validation to mitigate overfitting, this study has several limitations inherent to its exploratory design. First, the retrospective design of the cohort introduces inherent biases in data collection and ADR reporting. Second, despite restricting our main analysis to prevalent outcomes, the relatively small sample size (n = 178) combined with the evaluation of multiple genetic models inherently increases the risk of false-positive discoveries (Type I error). We chose not to apply a strict False Discovery Rate (FDR) correction to maintain the exploratory, hypothesis-generating nature of the study, but this limits the confirmatory power of the unadjusted *p*-values reported (Steyerberg E. W., et al., 2010).

Third, a crucial statistical consideration is the risk of optimism bias. Although we employed LASSO regularization and reported optimism-corrected AUCs via 1000-iteration bootstrapping to estimate robust internal validity, predictive models developed and evaluated within the same cohort are inherently susceptible to performance inflation. The internal metrics provided may still overestimate the true discriminatory capacity of the scores. The absence of an external validation cohort prevents us from determining the generalizability and calibration of these models in independent Chilean cohorts, in other Latin American populations, or in non-Latin American settings.

Finally, there is likely residual clinical confounding. While we controlled for basic demographic and anthropometric covariates (age and BSA), these models should be interpreted as partially adjusted associations rather than definitive estimates of independent pharmacogenetic effect. Although induction treatment followed a common IA/IA* protocol backbone, residual confounding by patient-level treatment exposure cannot be fully excluded. Individual dose modifications, treatment delays, supportive-care interventions, and pharmacokinetic exposure were not consistently available in this retrospective dataset and could not be directly modeled. Importantly, definitive post-induction risk reclassification was not incorporated as an adjustment covariate because it does not represent a baseline determinant of induction treatment intensity and may be informed by response parameters assessed during or after the induction window. Future studies should prospectively collect patient-level drug exposure, dose-modification, treatment-delay, and pharmacokinetic data to determine whether pharmacogenomic markers provide incremental predictive value beyond treatment exposure and established clinical factors (Collins et al., 2015). Because this analysis was restricted to induction, the findings should not be extrapolated to thiopurine-intensive treatment phases, where TPMT and NUDT15 have established clinical utility for mercaptopurine dosing. Conversely, TPMT/NUDT15 were not modeled as mandatory adjustment covariates for induction toxicity because their strongest mechanistic and clinical relevance depends on thiopurine exposure.

An additional methodological consideration is that a subset of loci showed deviation from HWE and/or sparse genotype categories. Therefore, the apparent inheritance pattern of individual associations should be interpreted cautiously and not as definitive evidence of the underlying biological mode of action. The analytical framework was intentionally retained within a single reproducible R-based workflow, integrating multivariable logistic regression, penalized selection, and bootstrap-based internal validation rather than splitting inference across different external platforms. Another limitation is that several prioritized gene–phenotype relationships were indirect or partially discordant with the phenotypes most consistently reported in previous pharmacogenetic literature. Therefore, these associations should be interpreted as candidate predictive markers requiring replication rather than as established causal mechanisms.

Future work should focus on (i) prospective multicenter validation, first in independent Chilean pediatric ALL cohorts and subsequently across broader Latin American populations, (ii) expansion of genetic coverage to include established loci for specific toxicities (e.g., HLA for asparaginase hypersensitivity), (iii) incorporation of richer, time-resolved toxicity endpoints, and (iv) integration of clinical predictors to build hybrid clinico-genomic models that can be evaluated under TRIPOD-aligned external validation frameworks.

## 5 Conclusion

The primary contribution of this study is the prioritization of pharmacogenomic candidate signals rather than the proposal of models ready for clinical intervention. The integration of alternative genotype parameterizations and regularized machine learning provides valuable exploratory insights into the pharmacogenomic landscape of ALL induction therapy. The extracted Pharmacogenetic Risk Scores (PGRS) for Neutropenia and Anemia offer parsimonious, multi-locus candidate-variant tools that underscore the potential contribution of metabolic enzymes (CYP3A5) and immune-related pathways (NFATC2, TLR2). However, given the retrospective nature of the study, the near-universal baseline prevalence of hematological toxicities, and the inherent risks of internal optimism, these scores serve strictly as biologically informed proof-of-concept models. Robust prospective external validation is categorically required before these genetic signatures can be considered to inform risk-adapted supportive care.

## Data Availability

The authors acknowledge that the data presented in this study must be deposited and made publicly available in an acceptable repository, prior to publication. Frontiers cannot accept a manuscript that does not adhere to our open data policies.
