# Supplementary material for: Pharmacogenomic impact and genetic architecture of toxicity in pediatric acute lymphoblastic leukemia induction therapy: an exploratory modeling approach
Source: Front Pharmacol. 2026 Jun 16;17:1830183. doi: 10.3389/fphar.2026.1830183 (PMC13314868; doi:10.3389/fphar.2026.1830183)
Supplement: Supplementary file 1 [file Supplementaryfile1.docx]

***Supplementary Material***

# Supplementary Data

**Supplementary Figure S1.** Overall study design and analytical workflow for pharmacogenomic risk score development in pediatric acute lymphoblastic leukemia (ALL) induction toxicity.

The flowchart summarizes the retrospective cohort design and source population, clinical phenotyping and ADR classification, genotyping and quality-control procedures, adjusted exploratory association analysis across alternative inheritance models, LASSO-based pharmacogenomic risk score (PGRS) construction, and internal validation using optimism-corrected AUC, 95% confidence intervals, Brier score, and Youden index.

**
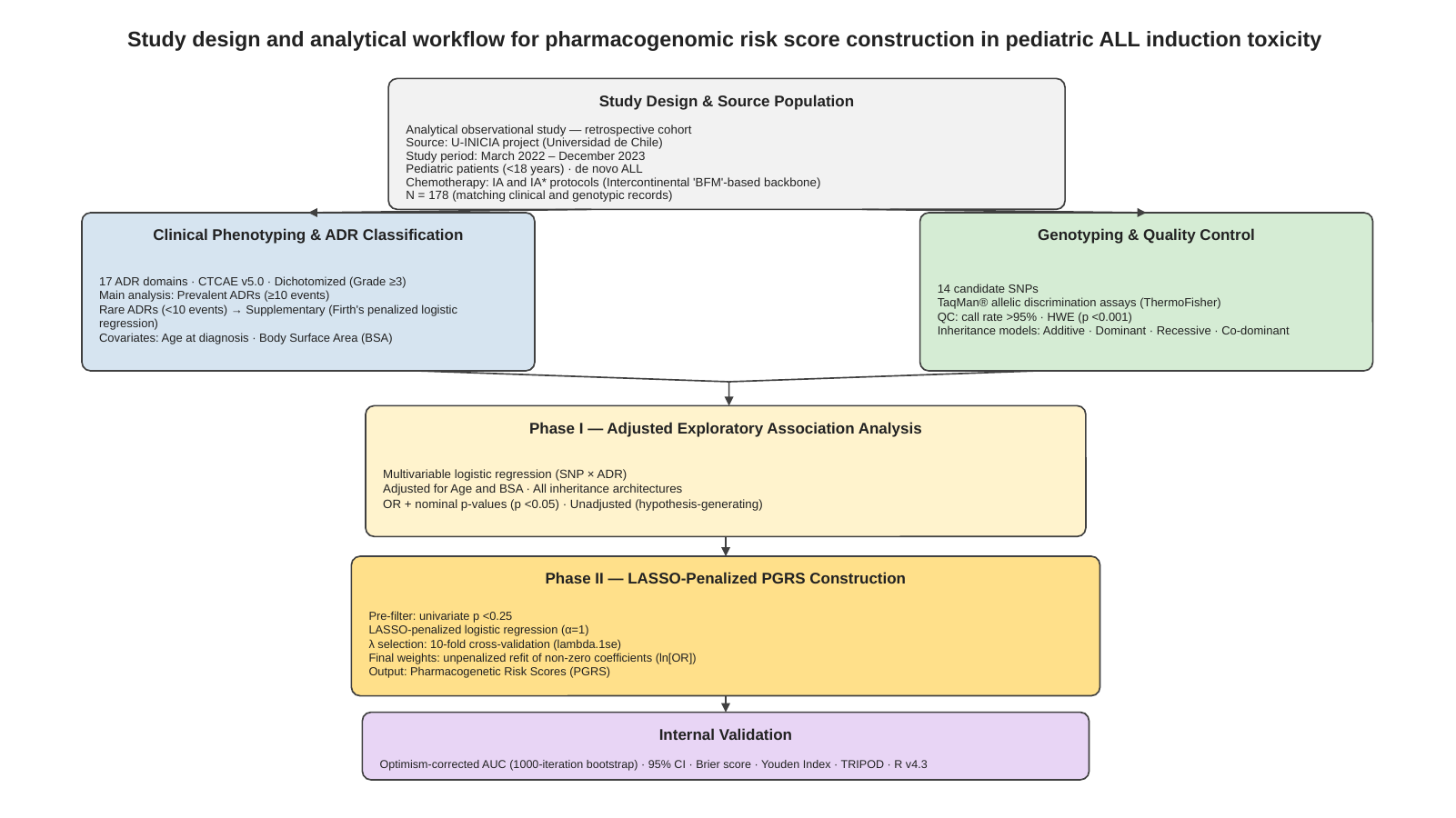
**

**Supplementary table S1**. Operational definitions of ADR phenotypes during induction therapy.

| ADR domain | Proposed operational definition for the binary endpoint | Objective grading rule | Source type | Coding rule |
| --- | --- | --- | --- | --- |
| Anemia | Presence of CTCAE v5.0 grade ≥3 anemia at any time during induction | Hemoglobin <8.0 g/dL and/or transfusion indicated; use the worst value/grade during induction | Laboratory-based | Binary coded as 1 if any grade ≥3 episode occurred during induction; otherwise 0 |
| Neutropenia | Presence of CTCAE v5.0 grade ≥3 neutrophil count decreased at any time during induction | ANC <1000/mm³ (grade 3: <1000 to 500/mm³; grade 4: <500/mm³); use the worst value/grade during induction | Laboratory-based | Coded independently from infection; a patient may be positive for both |
| Thrombocytopenia | Presence of CTCAE v5.0 grade ≥3 platelet count decreased at any time during induction | Platelets <50,000/mm³ (grade 3: <50,000 to 25,000/mm³; grade 4: <25,000/mm³); use the worst value/grade during induction | Laboratory-based | Binary coded from the worst platelet value during induction |
| Infection | Presence of a clinically documented infection meeting CTCAE-compatible grade ≥3 severity during induction | Suggested objective rule: infection requiring hospitalization and/or prolongation of hospitalization, IV antimicrobial therapy, and/or invasive intervention; use the worst documented infectious episode during induction | Clinical diagnosis from chart review | Coded independently from neutropenia. Infection occurring during neutropenia is counted in both domains if both criteria are met |
| Gastrointestinal toxicity | Presence of any prespecified CTCAE v5.0 GI event grade ≥3 during induction | Recommended composite definition: any of the following at grade ≥3: diarrhea (e.g., ≥7 stools/day over baseline or hospitalization indicated), nausea (inadequate oral caloric/fluid intake; tube feeding/TPN/hospitalization indicated), oral mucositis with grade ≥3 severity, GI bleeding, ileus/obstruction, or other prespecified GI CTCAE term; use the worst GI grade during induction | Mixed: laboratory/ clinical depending on term | Should be based on a closed predefined list of GI CTCAE terms to avoid post hoc classification |
| L-Asparaginase hypersensitivity | Presence of CTCAE v5.0 grade ≥3 allergic reaction/anaphylaxis temporally associated with L-asparaginase during induction | Suggested objective rule: physician-documented hypersensitivity reaction during or after L-asparaginase exposure with bronchospasm, angioedema, hypotension, parenteral/IV intervention, and/or hospitalization; retain the worst grade during induction | Clinical diagnosis from chart review | Count as one binary outcome regardless of number of episodes; do not double-code the same event as both “allergic reaction” and “anaphylaxis” within this domain |
| Electrolyte disorder | Presence of any prespecified CTCAE v5.0 electrolyte abnormality grade ≥3 during induction | Recommended composite definition: worst grade ≥3 among predefined electrolytes, for example hyponatremia, hypokalemia, hypocalcemia, hypomagnesemia, or hypophosphatemia. Suggested examples: hyponatremia 120–124 mmol/L regardless of symptoms or symptomatic 125–129 mmol/L; hypokalemia 2.5–3.0 mmol/L with hospitalization indicated; use the worst electrolyte grade during induction | Laboratory-based | Should be based on a closed predefined list of electrolytes and the worst laboratory grade during induction |

ADRs were graded according to CTCAE v5.0. For each domain, the highest grade recorded during the induction phase was retained. Laboratory-based toxicities were defined using CTCAE laboratory thresholds. Clinically defined events were based on physician-documented diagnoses and mapped to CTCAE-compatible grade ≥3 severity criteria. ADR domains were coded independently and were not mutually exclusive; therefore, overlapping events (e.g., infection during neutropenia) could contribute to more than one outcome.

**Supplementary Table S2.** Complete association analysis of genetic variants and treatment-related toxicities in patients with ALL (including non-significant results). In bold, statistically significant association.

| **Toxicity** | **genetic variant** | **Additive OR [95% CI] (p)** | **Dominant OR [95% CI] (p)** | **Recessive OR [95% CI] (p)** | **Co-dom Het OR [95% CI] (p)** | **Co-dom Hom OR [95% CI] (p)** |
| --- | --- | --- | --- | --- | --- | --- |
| Anaphylaxis | ABCB1 (rs1045642) | 0.57 [0.15-2.14] (p=0.406) | >100 [-] (p=1.000) | >100 [-] (p=1.000) | - | - |
| Anaphylaxis | CYP3A5 (rs4646450) | >100 [-] (p=1.000) | >100 [-] (p=1.000) | >100 [-] (p=1.000) | >100 [-] (p=1.000) | >100 [-] (p=1.000) |
| Anaphylaxis | CYP3A5 (rs776746) | 0.72 [0.18-2.91] (p=0.646) | >100 [-] (p=0.993) | 0.44 [0.07-2.78] (p=0.384) | >100 [-] (p=nan) | >100 [-] (p=nan) |
| Anaphylaxis | IL6 (rs1800796) | 0.68 [0.15-3.06] (p=0.618) | 0.24 [0.02-2.68] (p=0.244) | 1.05 [0.14-7.79] (p=0.961) | 0.16 [0.01-3.10] (p=0.227) | 0.30 [0.02-3.85] (p=0.354) |
| Anaphylaxis | MTHFR (rs181133) | 0.25 [0.03-2.27] (p=0.218) | 0.22 [0.02-2.78] (p=0.245) | >100 [-] (p=1.000) | 0.33 [0.03-3.99] (p=0.380) | >100 [-] (p=1.000) |
| Anaphylaxis | MTHFR (rs181131) | 1.28 [0.13-12.23] (p=0.828) | >100 [-] (p=1.000) | 1.20 [0.10-13.68] (p=0.885) | >100 [-] (p=0.999) | >100 [-] (p=0.999) |
| Anaphylaxis | NUDT15 (rs116855232) | 4.36 [0.41-46.19] (p=0.221) | 4.36 [0.41-46.19] (p=0.221) | - | - | - |
| Anaphylaxis | OAT4 (rs11231809) | 0.33 [0.07-1.53] (p=0.157) | **0.05 [0.00-0.43] (p=0.007)** | 0.96 [0.13-7.19] (p=0.972) | >100 [-] (p=1.000) | **0.09 [0.01-0.84] (p=0.035)** |
| Anaphylaxis | OATP1B1 (rs11045879) | 0.69 [0.14-3.33] (p=0.643) | >100 [-] (p=1.000) | 0.54 [0.08-3.47] (p=0.517) | >100 [-] (p=1.000) | >100 [-] (p=1.000) |
| Anaphylaxis | SLC28A2 (rs11854484) | 1.81 [0.30-10.95] (p=0.518) | - | >100 [-] (p=1.000) | - | - |
| Anaphylaxis | TLR2 (rs4696480) | >100 [-] (p=1.000) | >100 [-] (p=1.000) | - | - | - |
| Anaphylaxis | TPMT (rs1142345) | - | >100 [-] (p=0.999) | - | >100 [-] (p=1.000) | >100 [-] (p=1.000) |
| Anaphylaxis | TPMT (rs1800460) | >100 [-] (p=0.988) | - | >100 [-] (p=1.000) | >100 [-] (p=1.000) | >100 [-] (p=1.000) |
| Anaphylaxis | NFATC2 (rs6021191) | >100 [-] (p=1.000) | >100 [-] (p=1.000) | - | - | - |
| Metabolic/Endocrine | ABCB1 (rs1045642) | 0.60 [0.18-2.07] (p=0.421) | 0.30 [0.05-1.96] (p=0.209) | 0.85 [0.14-5.18] (p=0.857) | 0.26 [0.03-2.21] (p=0.219) | 0.35 [0.04-3.04] (p=0.341) |
| Metabolic/Endocrine | CYP3A5 (rs4646450) | >100 [-] (p=1.000) | >100 [-] (p=1.000) | >100 [-] (p=1.000) | >100 [-] (p=1.000) | >100 [-] (p=1.000) |
| Metabolic/Endocrine | CYP3A5 (rs776746) | 0.78 [0.18-3.38] (p=0.740) | - | 0.54 [0.09-3.36] (p=0.507) | >100 [-] (p=0.999) | >100 [-] (p=0.999) |
| Metabolic/Endocrine | IL6 (rs1800796) | >100 [-] (p=1.000) | >100 [-] (p=0.999) | >100 [-] (p=1.000) | >100 [-] (p=1.000) | >100 [-] (p=0.999) |
| Metabolic/Endocrine | MTHFR (rs181133) | 1.74 [0.45-6.76] (p=0.423) | >100 [-] (p=1.000) | 0.81 [0.08-8.44] (p=0.862) | >100 [-] (p=0.997) | >100 [-] (p=0.997) |
| Metabolic/Endocrine | MTHFR (rs181131) | 0.47 [0.10-2.15] (p=0.332) | >100 [-] (p=1.000) | 0.30 [0.04-2.10] (p=0.223) | >100 [-] (p=1.000) | >100 [-] (p=1.000) |
| Metabolic/Endocrine | NUDT15 (rs116855232) | >100 [-] (p=0.924) | >100 [-] (p=0.924) | - | - | - |
| Metabolic/Endocrine | OAT4 (rs11231809) | 4.60 [0.19-114.12] (p=0.351) | >100 [-] (p=1.000) | 2.21 [0.09-57.41] (p=0.633) | >100 [-] (p=0.999) | >100 [-] (p=0.999) |
| Metabolic/Endocrine | OATP1B1 (rs11045879) | 1.98 [0.21-18.81] (p=0.553) | >100 [-] (p=1.000) | 1.97 [0.19-20.10] (p=0.569) | >100 [-] (p=1.000) | >100 [-] (p=1.000) |
| Metabolic/Endocrine | SLC28A2 (rs11854484) | 2.66 [0.80-8.86] (p=0.111) | 3.36 [0.49-23.19] (p=0.219) | 5.01 [0.64-39.08] (p=0.124) | 2.20 [0.24-19.70] (p=0.482) | 7.16 [0.67-76.28] (p=0.103) |
| Metabolic/Endocrine | TLR2 (rs4696480) | >100 [-] (p=1.000) | >100 [-] (p=1.000) | - | - | - |
| Metabolic/Endocrine | TPMT (rs1142345) | - | >100 [-] (p=0.920) | >100 [-] (p=0.993) | >100 [-] (p=0.997) | >100 [-] (p=0.999) |
| Metabolic/Endocrine | TPMT (rs1800460) | 0.31 [0.03-3.66] (p=0.350) | - | 0.31 [0.03-3.66] (p=0.350) | >100 [-] (p=1.000) | >100 [-] (p=1.000) |
| Metabolic/Endocrine | NFATC2 (rs6021191) | 3.49 [0.29-42.18] (p=0.326) | 3.49 [0.29-42.18] (p=0.326) | - | - | - |
| Hepatobiliary | ABCB1 (rs1045642) | 0.77 [0.27-2.20] (p=0.619) | 1.10 [0.12-9.81] (p=0.932) | 0.54 [0.10-2.79] (p=0.460) | 1.42 [0.15-13.47] (p=0.761) | 0.71 [0.06-8.57] (p=0.789) |
| Hepatobiliary | CYP3A5 (rs4646450) | - | >100 [-] (p=1.000) | - | >100 [-] (p=1.000) | >100 [-] (p=1.000) |
| Hepatobiliary | CYP3A5 (rs776746) | 1.14 [0.28-4.56] (p=0.857) | 0.33 [0.03-3.18] (p=0.337) | 2.30 [0.26-20.63] (p=0.457) | >100 [-] (p=1.000) | 0.39 [0.04-3.84] (p=0.421) |
| Hepatobiliary | IL6 (rs1800796) | 1.27 [0.31-5.18] (p=0.736) | 0.35 [0.03-3.62] (p=0.381) | 2.25 [0.38-13.34] (p=0.374) | 0.13 [0.01-2.66] (p=0.188) | 0.55 [0.05-5.92] (p=0.626) |
| Hepatobiliary | MTHFR (rs181133) | **4.38 [1.14-16.84] (p=0.032)** | 2.84 [0.32-25.24] (p=0.350) | **10.58 [1.79-62.61] (p=0.009)** | 0.77 [0.05-12.73] (p=0.857) | 9.22 [0.95-89.35] (p=0.055) |
| Hepatobiliary | MTHFR (rs181131) | 1.33 [0.27-6.65] (p=0.726) | >100 [-] (p=1.000) | 1.24 [0.22-7.08] (p=0.805) | >100 [-] (p=1.000) | >100 [-] (p=1.000) |
| Hepatobiliary | NUDT15 (rs116855232) | >100 [-] (p=1.000) | >100 [-] (p=1.000) | - | - | - |
| Hepatobiliary | OAT4 (rs11231809) | 0.94 [0.27-3.31] (p=0.928) | >100 [-] (p=1.000) | 0.47 [0.08-2.68] (p=0.398) | >100 [-] (p=0.522) | >100 [-] (p=0.585) |
| Hepatobiliary | OATP1B1 (rs11045879) | 0.51 [0.15-1.66] (p=0.263) | 0.12 [0.01-1.34] (p=0.085) | 0.62 [0.14-2.84] (p=0.543) | 0.12 [0.01-2.03] (p=0.143) | 0.12 [0.01-1.36] (p=0.086) |
| Hepatobiliary | SLC28A2 (rs11854484) | 1.03 [0.37-2.84] (p=0.952) | 2.07 [0.45-9.54] (p=0.352) | >100 [-] (p=0.999) | 3.16 [0.67-14.84] (p=0.145) | >100 [-] (p=0.999) |
| Hepatobiliary | TLR2 (rs4696480) | >100 [-] (p=1.000) | >100 [-] (p=1.000) | - | - | - |
| Hepatobiliary | TPMT (rs1142345) | 0.75 [0.13-4.41] (p=0.752) | >100 [-] (p=0.998) | 0.49 [0.05-4.57] (p=0.535) | >100 [-] (p=0.999) | >100 [-] (p=0.999) |
| Hepatobiliary | TPMT (rs1800460) | 0.50 [0.05-4.57] (p=0.538) | - | 0.50 [0.05-4.57] (p=0.538) | >100 [-] (p=1.000) | >100 [-] (p=1.000) |
| Hepatobiliary | NFATC2 (rs6021191) | >100 [-] (p=1.000) | >100 [-] (p=1.000) | - | - | - |
| Neutropenia | ABCB1 (rs1045642) | 0.51 [0.19-1.37] (p=0.183) | >100 [-] (p=1.000) | 0.58 [0.18-1.90] (p=0.369) | >100 [-] (p=1.000) | >100 [-] (p=1.000) |
| Neutropenia | CYP3A5 (rs4646450) | >100 [-] (p=1.000) | >100 [-] (p=1.000) | >100 [-] (p=1.000) | >100 [-] (p=1.000) | >100 [-] (p=1.000) |
| Neutropenia | CYP3A5 (rs776746) | **2.59 [1.14-5.85] (p=0.022)** | 1.66 [0.19-14.63] (p=0.647) | **5.58 [1.62-19.19] (p=0.006)** | 0.49 [0.05-4.77] (p=0.542) | 3.16 [0.32-30.86] (p=0.323) |
| Neutropenia | IL6 (rs1800796) | 1.28 [0.41-4.03] (p=0.674) | >100 [-] (p=0.982) | 2.38 [0.44-12.84] (p=0.314) | >100 [-] (p=1.000) | >100 [-] (p=1.000) |
| Neutropenia | MTHFR (rs181133) | 0.41 [0.07-2.37] (p=0.317) | >100 [-] (p=0.999) | 0.53 [0.04-6.53] (p=0.618) | 0.37 [0.01-9.17] (p=0.544) | 0.32 [0.01-10.85] (p=0.525) |
| Neutropenia | MTHFR (rs181131) | 0.84 [0.09-8.00] (p=0.879) | >100 [-] (p=1.000) | 0.92 [0.08-10.97] (p=0.948) | >100 [-] (p=1.000) | >100 [-] (p=1.000) |
| Neutropenia | NUDT15 (rs116855232) | >100 [-] (p=1.000) | >100 [-] (p=1.000) | - | - | - |
| Neutropenia | OAT4 (rs11231809) | 1.27 [0.37-4.31] (p=0.703) | 1.73 [0.15-20.42] (p=0.662) | 1.21 [0.25-5.87] (p=0.812) | 1.65 [0.13-21.55] (p=0.705) | 1.84 [0.13-25.36] (p=0.649) |
| Neutropenia | OATP1B1 (rs11045879) | 0.30 [0.04-2.35] (p=0.253) | >100 [-] (p=1.000) | 0.29 [0.04-2.42] (p=0.254) | >100 [-] (p=0.999) | >100 [-] (p=0.999) |
| Neutropenia | SLC28A2 (rs11854484) | 0.73 [0.23-2.35] (p=0.596) | 0.59 [0.09-3.77] (p=0.576) | 0.71 [0.07-6.77] (p=0.763) | 0.60 [0.08-4.59] (p=0.624) | 0.56 [0.05-6.74] (p=0.649) |
| Neutropenia | TLR2 (rs4696480) | >100 [-] (p=0.999) | >100 [-] (p=0.999) | - | - | - |
| Neutropenia | TPMT (rs1142345) | - | >100 [-] (p=0.999) | - | >100 [-] (p=1.000) | >100 [-] (p=1.000) |
| Neutropenia | TPMT (rs1800460) | 1.23 [0.13-11.33] (p=0.855) | - | 1.23 [0.13-11.33] (p=0.855) | 2.76 [nan-nan] (p=nan) | 3.39 [nan-nan] (p=nan) |
| Neutropenia | NFATC2 (rs6021191) | **0.09 [0.02-0.42] (p=0.002)** | **0.09 [0.02-0.42] (p=0.002)** | - | - | - |
| Anemia | ABCB1 (rs1045642) | 0.58 [0.18-1.94] (p=0.378) | - | 0.70 [0.16-3.06] (p=0.633) | >100 [-] (p=0.999) | >100 [-] (p=0.999) |
| Anemia | CYP3A5 (rs4646450) | >100 [-] (p=1.000) | >100 [-] (p=1.000) | >100 [-] (p=1.000) | >100 [-] (p=1.000) | >100 [-] (p=1.000) |
| Anemia | CYP3A5 (rs776746) | 2.50 [0.93-6.73] (p=0.069) | 2.77 [0.28-26.89] (p=0.381) | 4.52 [0.97-21.01] (p=0.054) | 0.98 [0.09-11.07] (p=0.987) | 4.46 [0.41-48.93] (p=0.222) |
| Anemia | IL6 (rs1800796) | 0.91 [0.21-3.85] (p=0.893) | >100 [-] (p=0.999) | 1.25 [0.19-8.19] (p=0.813) | >100 [-] (p=1.000) | >100 [-] (p=1.000) |
| Anemia | MTHFR (rs181133) | 1.24 [0.20-7.49] (p=0.817) | 1.23 [0.09-17.47] (p=0.881) | 1.63 [0.04-74.70] (p=0.801) | 1.06 [0.06-19.27] (p=0.966) | 1.68 [0.03-91.72] (p=0.800) |
| Anemia | MTHFR (rs181131) | 1.12 [0.09-13.73] (p=0.928) | >100 [-] (p=1.000) | 1.20 [0.08-16.96] (p=0.892) | >100 [-] (p=1.000) | >100 [-] (p=1.000) |
| Anemia | NUDT15 (rs116855232) | >100 [-] (p=0.999) | >100 [-] (p=0.999) | - | - | - |
| Anemia | OAT4 (rs11231809) | 0.66 [0.13-3.38] (p=0.617) | >100 [-] (p=1.000) | 0.73 [0.12-4.56] (p=0.733) | >100 [-] (p=1.000) | >100 [-] (p=1.000) |
| Anemia | OATP1B1 (rs11045879) | 0.52 [0.06-4.37] (p=0.550) | >100 [-] (p=1.000) | 0.53 [0.05-5.19] (p=0.588) | >100 [-] (p=0.999) | >100 [-] (p=0.999) |
| Anemia | SLC28A2 (rs11854484) | 2.92 [0.37-23.22] (p=0.312) | 3.06 [0.29-31.73] (p=0.349) | >100 [-] (p=1.000) | 2.24 [0.21-23.55] (p=0.500) | >100 [-] (p=1.000) |
| Anemia | TLR2 (rs4696480) | >100 [-] (p=0.993) | >100 [-] (p=0.993) | - | - | - |
| Anemia | TPMT (rs1142345) | >100 [-] (p=0.999) | >100 [-] (p=0.999) | >100 [-] (p=0.999) | >100 [-] (p=1.000) | >100 [-] (p=0.998) |
| Anemia | TPMT (rs1800460) | 2.91 [0.30-28.12] (p=0.356) | - | 2.91 [0.30-28.12] (p=0.356) | >100 [-] (p=1.000) | >100 [-] (p=1.000) |
| Anemia | NFATC2 (rs6021191) | **0.15 [0.03-0.83] (p=0.030)** | **0.15 [0.03-0.83] (p=0.030)** | - | - | - |
| Neurotoxicity | ABCB1 (rs1045642) | 2.54 [0.66-9.74] (p=0.176) | >100 [-] (p=1.000) | 2.44 [0.51-11.63] (p=0.264) | >100 [-] (p=1.000) | >100 [-] (p=1.000) |
| Neurotoxicity | CYP3A5 (rs776746) | 0.97 [0.24-3.90] (p=0.962) | >100 [-] (p=0.999) | 0.73 [0.13-4.03] (p=0.714) | >100 [-] (p=nan) | >100 [-] (p=nan) |
| Neurotoxicity | IL6 (rs1800796) | >100 [-] (p=1.000) | >100 [-] (p=0.999) | >100 [-] (p=1.000) | >100 [-] (p=1.000) | >100 [-] (p=1.000) |
| Neurotoxicity | MTHFR (rs181133) | 2.92 [0.71-12.10] (p=0.139) | >100 [-] (p=0.970) | 2.56 [0.38-17.39] (p=0.336) | >100 [-] (p=1.000) | >100 [-] (p=1.000) |
| Neurotoxicity | MTHFR (rs181131) | - | >100 [-] (p=1.000) | >100 [-] (p=0.999) | >100 [-] (p=1.000) | >100 [-] (p=0.999) |
| Neurotoxicity | NUDT15 (rs116855232) | >100 [-] (p=1.000) | >100 [-] (p=1.000) | - | - | - |
| Neurotoxicity | OAT4 (rs11231809) | 1.98 [0.39-10.10] (p=0.410) | 0.37 [0.03-4.03] (p=0.413) | 5.29 [0.54-51.35] (p=0.151) | >100 [-] (p=0.876) | 0.81 [0.07-9.38] (p=0.863) |
| Neurotoxicity | OATP1B1 (rs11045879) | 0.89 [0.18-4.31] (p=0.885) | >100 [-] (p=1.000) | 0.77 [0.13-4.54] (p=0.774) | >100 [-] (p=0.999) | >100 [-] (p=0.999) |
| Neurotoxicity | SLC28A2 (rs11854484) | **9.08 [1.65-50.05] (p=0.011)** | - | **14.50 [1.76-119.49] (p=0.013)** | >100 [-] (p=nan) | >100 [-] (p=nan) |
| Neurotoxicity | TLR2 (rs4696480) | 0.55 [0.07-4.25] (p=0.564) | 0.55 [0.07-4.25] (p=0.564) | - | - | - |
| Neurotoxicity | TPMT (rs1142345) | - | >100 [-] (p=0.998) | - | >100 [-] (p=0.694) | >100 [-] (p=0.833) |
| Neurotoxicity | TPMT (rs1800460) | 0.33 [0.03-3.34] (p=0.349) | - | 0.33 [0.03-3.34] (p=0.349) | >100 [-] (p=1.000) | >100 [-] (p=1.000) |
| Neurotoxicity | NFATC2 (rs6021191) | >100 [-] (p=0.996) | >100 [-] (p=0.996) | - | - | - |
| Thrombosis | ABCB1 (rs1045642) | 1.94 [0.49-7.63] (p=0.345) | - | 1.69 [0.32-8.90] (p=0.534) | >100 [-] (p=1.000) | >100 [-] (p=1.000) |
| Thrombosis | CYP3A5 (rs4646450) | >100 [-] (p=1.000) | - | >100 [-] (p=1.000) | - | - |
| Thrombosis | CYP3A5 (rs776746) | 0.83 [0.21-3.33] (p=0.792) | >100 [-] (p=0.999) | 0.57 [0.10-3.31] (p=0.529) | >100 [-] (p=1.000) | >100 [-] (p=1.000) |
| Thrombosis | IL6 (rs1800796) | >100 [-] (p=0.999) | >100 [-] (p=0.999) | >100 [-] (p=1.000) | >100 [-] (p=1.000) | >100 [-] (p=1.000) |
| Thrombosis | MTHFR (rs181133) | 1.93 [0.52-7.15] (p=0.324) | >100 [-] (p=1.000) | 0.95 [0.10-9.39] (p=0.967) | >100 [-] (p=0.999) | >100 [-] (p=0.999) |
| Thrombosis | MTHFR (rs181131) | 2.38 [0.28-20.37] (p=0.427) | >100 [-] (p=1.000) | 2.39 [0.25-22.47] (p=0.445) | >100 [-] (p=1.000) | >100 [-] (p=1.000) |
| Thrombosis | NUDT15 (rs116855232) | >100 [-] (p=0.999) | >100 [-] (p=0.999) | - | - | - |
| Thrombosis | OAT4 (rs11231809) | 1.45 [0.27-7.80] (p=0.662) | 0.27 [0.02-3.23] (p=0.304) | 3.87 [0.37-40.36] (p=0.258) | >100 [-] (p=0.985) | 0.59 [0.05-7.34] (p=0.680) |
| Thrombosis | OATP1B1 (rs11045879) | 0.67 [0.14-3.20] (p=0.616) | >100 [-] (p=1.000) | 0.52 [0.08-3.32] (p=0.487) | >100 [-] (p=0.995) | >100 [-] (p=0.995) |
| Thrombosis | SLC28A2 (rs11854484) | **5.83 [1.15-29.61] (p=0.033)** | >100 [-] (p=0.981) | 7.23 [0.86-60.90] (p=0.069) | >100 [-] (p=nan) | >100 [-] (p=nan) |
| Thrombosis | TLR2 (rs4696480) | 1.83 [0.18-18.40] (p=0.609) | 1.83 [0.18-18.40] (p=0.609) | - | - | - |
| Thrombosis | TPMT (rs1142345) | >100 [-] (p=0.975) | >100 [-] (p=1.000) | >100 [-] (p=0.996) | >100 [-] (p=0.975) | >100 [-] (p=0.999) |
| Thrombosis | TPMT (rs1800460) | 0.38 [0.04-4.01] (p=0.424) | - | 0.38 [0.04-4.01] (p=0.424) | 0.41 [nan-nan] (p=nan) | 0.16 [nan-nan] (p=nan) |
| Thrombosis | NFATC2 (rs6021191) | 1.89 [0.19-18.79] (p=0.589) | 1.89 [0.19-18.79] (p=0.589) | - | - | - |
| L-Asparaginase Reac. | ABCB1 (rs1045642) | 0.91 [0.48-1.73] (p=0.783) | 1.01 [0.27-3.73] (p=0.993) | 0.85 [0.35-2.03] (p=0.708) | 1.08 [0.28-4.23] (p=0.907) | 0.90 [0.22-3.74] (p=0.890) |
| L-Asparaginase Reac. | CYP3A5 (rs4646450) | 0.81 [0.05-12.71] (p=0.882) | >100 [-] (p=0.705) | 0.04 [0.00-44.14] (p=0.367) | >100 [-] (p=1.000) | >100 [-] (p=1.000) |
| L-Asparaginase Reac. | CYP3A5 (rs776746) | **0.50 [0.27-0.93] (p=0.029)** | **0.26 [0.07-0.96] (p=0.043)** | 0.44 [0.18-1.07] (p=0.069) | 0.39 [0.08-1.78] (p=0.223) | **0.23 [0.06-0.87] (p=0.031)** |
| L-Asparaginase Reac. | IL6 (rs1800796) | 1.88 [0.85-4.13] (p=0.118) | 2.54 [0.31-20.67] (p=0.383) | 2.10 [0.81-5.41] (p=0.125) | 1.72 [0.19-15.43] (p=0.627) | 3.31 [0.40-27.73] (p=0.269) |
| L-Asparaginase Reac. | MTHFR (rs181133) | 1.37 [0.70-2.66] (p=0.358) | 1.38 [0.49-3.85] (p=0.541) | 1.71 [0.55-5.31] (p=0.350) | 1.20 [0.40-3.62] (p=0.749) | 1.90 [0.51-7.06] (p=0.335) |
| L-Asparaginase Reac. | MTHFR (rs181131) | 1.63 [0.63-4.25] (p=0.317) | >100 [-] (p=1.000) | 1.56 [0.56-4.33] (p=0.395) | >100 [-] (p=1.000) | >100 [-] (p=1.000) |
| L-Asparaginase Reac. | NUDT15 (rs116855232) | 1.88 [0.47-7.45] (p=0.370) | 1.88 [0.47-7.45] (p=0.370) | - | - | - |
| L-Asparaginase Reac. | OAT4 (rs11231809) | 1.09 [0.50-2.37] (p=0.837) | 0.57 [0.11-3.06] (p=0.513) | 1.32 [0.51-3.40] (p=0.567) | 0.46 [0.08-2.71] (p=0.389) | 0.68 [0.12-3.84] (p=0.664) |
| L-Asparaginase Reac. | OATP1B1 (rs11045879) | 1.90 [0.66-5.44] (p=0.234) | >100 [-] (p=1.000) | 1.85 [0.59-5.77] (p=0.292) | 0.00 [nan-nan] (p=nan) | 0.00 [nan-nan] (p=nan) |
| L-Asparaginase Reac. | SLC28A2 (rs11854484) | 0.89 [0.46-1.74] (p=0.737) | 0.64 [0.24-1.69] (p=0.366) | 1.42 [0.42-4.85] (p=0.575) | 0.45 [0.13-1.51] (p=0.197) | 1.09 [0.30-3.88] (p=0.899) |
| L-Asparaginase Reac. | TLR2 (rs4696480) | 1.22 [0.45-3.32] (p=0.698) | 1.22 [0.45-3.32] (p=0.698) | - | - | - |
| L-Asparaginase Reac. | TPMT (rs1142345) | - | >100 [-] (p=0.999) | >100 [-] (p=1.000) | >100 [-] (p=0.999) | >100 [-] (p=0.999) |
| L-Asparaginase Reac. | TPMT (rs1800460) | 2.33 [0.27-20.22] (p=0.444) | - | 2.33 [0.27-20.22] (p=0.444) | 0.32 [nan-nan] (p=nan) | 0.73 [nan-nan] (p=nan) |
| L-Asparaginase Reac. | NFATC2 (rs6021191) | 2.49 [0.86-7.21] (p=0.093) | 2.49 [0.86-7.21] (p=0.093) | - | - | - |
| Infection | ABCB1 (rs1045642) | 1.24 [0.75-2.05] (p=0.400) | 1.07 [0.38-3.00] (p=0.903) | 1.41 [0.74-2.72] (p=0.298) | 0.91 [0.31-2.67] (p=0.860) | 1.31 [0.44-3.92] (p=0.634) |
| Infection | CYP3A5 (rs4646450) | 5.03 [0.38-67.32] (p=0.222) | >100 [-] (p=1.000) | 5.34 [0.36-79.22] (p=0.224) | >100 [-] (p=1.000) | >100 [-] (p=1.000) |
| Infection | CYP3A5 (rs776746) | 0.97 [0.56-1.66] (p=0.906) | 0.97 [0.27-3.52] (p=0.963) | 0.95 [0.45-1.99] (p=0.894) | 1.01 [0.24-4.30] (p=0.987) | 0.96 [0.26-3.52] (p=0.950) |
| Infection | IL6 (rs1800796) | 0.72 [0.42-1.22] (p=0.221) | 1.08 [0.33-3.48] (p=0.903) | 0.53 [0.26-1.10] (p=0.088) | 1.57 [0.45-5.41] (p=0.477) | 0.77 [0.22-2.64] (p=0.672) |
| Infection | MTHFR (rs181133) | 0.96 [0.57-1.61] (p=0.882) | 1.15 [0.54-2.44] (p=0.712) | 0.67 [0.24-1.88] (p=0.445) | 1.31 [0.59-2.89] (p=0.505) | 0.78 [0.25-2.40] (p=0.662) |
| Infection | MTHFR (rs181131) | 1.14 [0.59-2.23] (p=0.693) | >100 [-] (p=1.000) | 0.99 [0.47-2.07] (p=0.981) | >100 [-] (p=1.000) | >100 [-] (p=1.000) |
| Infection | NUDT15 (rs116855232) | 0.75 [0.22-2.61] (p=0.655) | 0.75 [0.22-2.61] (p=0.655) | - | - | - |
| Infection | OAT4 (rs11231809) | 0.86 [0.46-1.59] (p=0.624) | 0.27 [0.06-1.18] (p=0.082) | 1.12 [0.52-2.38] (p=0.772) | 0.23 [0.05-1.06] (p=0.060) | 0.32 [0.07-1.43] (p=0.136) |
| Infection | OATP1B1 (rs11045879) | 1.22 [0.63-2.36] (p=0.556) | 2.31 [0.25-21.48] (p=0.462) | 1.17 [0.54-2.55] (p=0.691) | 2.19 [0.21-22.41] (p=0.509) | 2.34 [0.25-21.86] (p=0.456) |
| Infection | SLC28A2 (rs11854484) | 1.14 [0.67-1.96] (p=0.622) | 1.07 [0.48-2.39] (p=0.863) | 1.49 [0.53-4.25] (p=0.451) | 0.92 [0.37-2.28] (p=0.857) | 1.45 [0.48-4.36] (p=0.514) |
| Infection | TLR2 (rs4696480) | 0.46 [0.21-1.01] (p=0.052) | 0.46 [0.21-1.01] (p=0.052) | - | - | - |
| Infection | TPMT (rs1142345) | 1.19 [0.45-3.17] (p=0.725) | >100 [-] (p=0.998) | 0.96 [0.30-3.14] (p=0.953) | >100 [-] (p=0.999) | >100 [-] (p=0.999) |
| Infection | TPMT (rs1800460) | 1.52 [0.39-5.98] (p=0.550) | - | 1.52 [0.39-5.98] (p=0.550) | 0.77 [nan-nan] (p=nan) | 1.17 [nan-nan] (p=nan) |
| Infection | NFATC2 (rs6021191) | 1.10 [0.45-2.72] (p=0.835) | 1.10 [0.45-2.72] (p=0.835) | - | - | - |
| Thrombocytopenia | ABCB1 (rs1045642) | 1.29 [0.74-2.28] (p=0.371) | 1.36 [0.46-4.07] (p=0.577) | 1.40 [0.64-3.08] (p=0.405) | 1.21 [0.39-3.77] (p=0.744) | 1.63 [0.49-5.44] (p=0.427) |
| Thrombocytopenia | CYP3A5 (rs4646450) | 1.14 [0.14-9.16] (p=0.902) | >100 [-] (p=1.000) | 1.73 [0.13-23.79] (p=0.682) | >100 [-] (p=1.000) | >100 [-] (p=1.000) |
| Thrombocytopenia | CYP3A5 (rs776746) | 1.04 [0.55-1.97] (p=0.898) | 0.39 [0.05-3.21] (p=0.384) | 1.33 [0.58-3.07] (p=0.500) | 0.26 [0.03-2.35] (p=0.230) | 0.44 [0.05-3.65] (p=0.451) |
| Thrombocytopenia | IL6 (rs1800796) | 0.95 [0.49-1.85] (p=0.885) | 0.68 [0.14-3.34] (p=0.637) | 1.05 [0.43-2.54] (p=0.922) | 0.64 [0.12-3.36] (p=0.594) | 0.72 [0.14-3.76] (p=0.700) |
| Thrombocytopenia | MTHFR (rs181133) | 1.30 [0.69-2.47] (p=0.415) | 1.33 [0.54-3.26] (p=0.536) | 1.63 [0.44-5.99] (p=0.465) | 1.20 [0.47-3.11] (p=0.701) | 1.80 [0.44-7.32] (p=0.410) |
| Thrombocytopenia | MTHFR (rs181131) | 0.99 [0.45-2.22] (p=0.990) | 1.67 [0.17-16.83] (p=0.662) | 0.93 [0.38-2.29] (p=0.870) | 1.81 [0.17-19.77] (p=0.625) | 1.60 [0.16-16.46] (p=0.693) |
| Thrombocytopenia | NUDT15 (rs116855232) | 0.76 [0.20-2.97] (p=0.698) | 0.76 [0.20-2.97] (p=0.698) | - | - | - |
| Thrombocytopenia | OAT4 (rs11231809) | 1.28 [0.63-2.62] (p=0.497) | 1.47 [0.28-7.74] (p=0.653) | 1.32 [0.54-3.23] (p=0.543) | 1.30 [0.23-7.27] (p=0.768) | 1.65 [0.29-9.36] (p=0.569) |
| Thrombocytopenia | OATP1B1 (rs11045879) | 1.47 [0.74-2.94] (p=0.274) | 2.98 [0.48-18.72] (p=0.243) | 1.43 [0.61-3.34] (p=0.409) | 2.51 [0.35-17.94] (p=0.361) | 3.13 [0.49-19.85] (p=0.226) |
| Thrombocytopenia | SLC28A2 (rs11854484) | 1.03 [0.55-1.90] (p=0.933) | 0.90 [0.36-2.24] (p=0.819) | 1.38 [0.37-5.22] (p=0.634) | 0.78 [0.29-2.11] (p=0.630) | 1.25 [0.31-5.05] (p=0.756) |
| Thrombocytopenia | TLR2 (rs4696480) | **0.29 [0.09-0.92] (p=0.036)** | **0.29 [0.09-0.92] (p=0.036)** | - | - | - |
| Thrombocytopenia | TPMT (rs1142345) | 0.35 [0.05-2.29] (p=0.273) | >100 [-] (p=0.999) | 0.33 [0.04-2.54] (p=0.287) | >100 [-] (p=0.998) | >100 [-] (p=0.998) |
| Thrombocytopenia | TPMT (rs1800460) | 1.47 [0.38-5.72] (p=0.581) | - | 1.47 [0.38-5.72] (p=0.581) | >100 [-] (p=1.000) | >100 [-] (p=1.000) |
| Thrombocytopenia | NFATC2 (rs6021191) | 1.24 [0.39-3.92] (p=0.720) | 1.24 [0.39-3.92] (p=0.720) | - | - | - |
| Gastrointestinal | ABCB1 (rs1045642) | 1.13 [0.61-2.10] (p=0.702) | 0.53 [0.17-1.60] (p=0.259) | 1.67 [0.75-3.72] (p=0.214) | 0.36 [0.11-1.23] (p=0.104) | 0.77 [0.24-2.50] (p=0.664) |
| Gastrointestinal | CYP3A5 (rs4646450) | - | >100 [-] (p=1.000) | - | 0.00 [nan-nan] (p=nan) | >100 [-] (p=nan) |
| Gastrointestinal | CYP3A5 (rs776746) | 0.89 [0.46-1.73] (p=0.734) | 0.52 [0.13-2.10] (p=0.357) | 1.03 [0.41-2.64] (p=0.943) | 0.40 [0.07-2.15] (p=0.283) | 0.55 [0.13-2.26] (p=0.408) |
| Gastrointestinal | IL6 (rs1800796) | 0.83 [0.44-1.59] (p=0.582) | 0.76 [0.19-2.98] (p=0.696) | 0.80 [0.34-1.92] (p=0.621) | 0.84 [0.20-3.56] (p=0.813) | 0.70 [0.17-2.94] (p=0.624) |
| Gastrointestinal | MTHFR (rs181133) | 1.19 [0.63-2.28] (p=0.589) | 1.35 [0.51-3.59] (p=0.546) | 1.15 [0.35-3.83] (p=0.814) | 1.34 [0.48-3.77] (p=0.574) | 1.37 [0.36-5.29] (p=0.648) |
| Gastrointestinal | MTHFR (rs181131) | 0.86 [0.38-1.94] (p=0.709) | 0.52 [0.05-5.40] (p=0.588) | 0.90 [0.35-2.27] (p=0.816) | 0.54 [0.05-6.04] (p=0.620) | 0.51 [0.05-5.43] (p=0.580) |
| Gastrointestinal | NUDT15 (rs116855232) | >100 [-] (p=0.999) | >100 [-] (p=0.999) | - | - | - |
| Gastrointestinal | OAT4 (rs11231809) | 0.57 [0.28-1.20] (p=0.140) | 0.32 [0.07-1.46] (p=0.142) | 0.60 [0.24-1.54] (p=0.287) | 0.38 [0.08-1.86] (p=0.235) | 0.27 [0.05-1.33] (p=0.107) |
| Gastrointestinal | OATP1B1 (rs11045879) | 1.11 [0.49-2.52] (p=0.800) | 0.83 [0.09-7.70] (p=0.866) | 1.20 [0.45-3.20] (p=0.712) | 0.68 [0.06-7.45] (p=0.750) | 0.86 [0.09-8.09] (p=0.896) |
| Gastrointestinal | SLC28A2 (rs11854484) | 0.92 [0.46-1.85] (p=0.824) | 1.37 [0.50-3.77] (p=0.545) | 0.27 [0.03-2.21] (p=0.225) | 1.97 [0.68-5.70] (p=0.212) | 0.37 [0.04-3.17] (p=0.363) |
| Gastrointestinal | TLR2 (rs4696480) | 2.41 [0.82-7.13] (p=0.111) | 2.41 [0.82-7.13] (p=0.111) | - | - | - |
| Gastrointestinal | TPMT (rs1142345) | 2.68 [0.39-18.24] (p=0.314) | >100 [-] (p=0.999) | 2.80 [0.35-22.46] (p=0.333) | >100 [-] (p=0.784) | >100 [-] (p=0.742) |
| Gastrointestinal | TPMT (rs1800460) | 2.46 [0.30-19.82] (p=0.399) | - | 2.46 [0.30-19.82] (p=0.399) | 0.33 [nan-nan] (p=nan) | 0.81 [nan-nan] (p=nan) |
| Gastrointestinal | NFATC2 (rs6021191) | 1.04 [0.33-3.32] (p=0.946) | 1.04 [0.33-3.32] (p=0.946) | - | - | - |

Supplementary table S3**. Exploratory Nominally Significant Associations for Prevalent ADRs Adjusted for Covariates (**p < 0.05**)**

| **Target ADR** | **Susceptibility Gene** | **Genetic Model (Comparison)*** | **Variant OR**  **95% CI (P-Value)** | **Age OR**  **95% CI (P-Value)** | **BSA OR**  **95% CI (P-Value)** |
| --- | --- | --- | --- | --- | --- |
| Neutropenia | NFATC2 (rs6021191) | Dominant (AG+GG vs AA) | 0.09  0.02-0.42 (p=0.0017) | 0.81  0.66-0.98 (p=0.0331) | 1.00  0.94-1.07 (p=0.9093) |
| Neutropenia | CYP3A5 (rs776746) | Recessive (GG vs AA+AG) | 5.58  1.62-19.19 (p=0.0063) | 0.88  0.75-1.03 (p=0.1145) | 1.00  0.94-1.06 (p=0.9908) |
| L-Asparaginase Reac. | CYP3A5 (rs776746) | Co-dom Homoz (GG vs AA) | 0.22  0.06-0.87 (p=0.0296) | 1.05  0.91-1.21 (p=0.4818) | 0.99  0.94-1.05 (p=0.8388) |
| Thrombocytopenia | TLR2 (rs4696480) | Dominant (TA+AA vs TT) | 0.29  0.09-0.92 (p=0.0188) | 0.99  0.86-1.14 (p=0.9084) | 0.96  0.89-1.05 (p=0.4075) |
| Infection | TLR2 (rs4696480) | Additive (Per 'A' allele) | 0.48  0.22-0.95 (p=0.0423) | 0.79  0.68-0.92 (p=0.0024) | 1.00  0.93-1.07 (p=0.9170) |

*Note: Additive models assess the continuous log-odds change per each additional variant allele. Dominant models compare any presence of the variant allele against the wild-type reference. Recessive models isolate the homozygous variant phenotype. Co-dominant models test the isolated effect of the homozygous variant against the wild-type reference without assuming linearity. In Figure 1, we use the protective variants/genotypes as reference to improve interpretability.*

Supplementary Table S4. Drug–gene–toxicity concordance matrix for prioritized pharmacogenetic signals identified during induction therapy in pediatric acute lymphoblastic leukemia

| Variant | Gene functional category | Variant type / genomic context | Drug(s) or treatment component biologically linked to the gene | Literature-supported mechanistic rationale | Observed toxicity phenotype in the present study | Concordance between literature and observed phenotype | Interpretation strength |
| --- | --- | --- | --- | --- | --- | --- | --- |
| CYP3A5 rs776746 | Drug metabolism | Functional splice-related variant; CYP3A5*3 low/non-expresser allele | Vincristine; broader induction exposure context including corticosteroids | CYP3A5 contributes substantially to vincristine metabolism, and rs776746 is the canonical reduced-expression allele. Prior literature more strongly supports a link with altered vincristine disposition and neurotoxicity than with direct hematologic toxicity. | Severe neutropenia; inverse signal for L-asparaginase hypersensitivity in exploratory models. | Indirect / exploratory concordance | Moderate biological plausibility; limited phenotype-specific support |
| NFATC22 rs6021191 | Immune regulation / transcriptional control | Intronic regulatory variant; eQTL-like signal | Immune response to asparaginase and treatment-related inflammatory reactivity | rs6021191 is an intronic variant associated with higher NFATC22 expression and increased risk of asparaginase hypersensitivity in pediatric ALL, supporting an immune-regulatory rather than truncating/protein-disrupting mechanism. | Exploratory hematologic/infection-related signals in the present study; interpret according to the final harmonized results table and discussion. | Indirect concordance | Exploratory |
| TLR2 rs4696480 | Innate immune signaling | Promoter/non-coding regulatory variant (T-16934A) | Host inflammatory response under chemotherapy-induced stress; susceptibility to infectious/inflammatory complications | rs4696480 is a non-coding promoter variant linked to altered innate immune responsiveness; variant carriers have shown reduced cytokine upregulation after TLR2 stimulation in functional studies, supporting a regulatory inflammatory mechanism rather than a truncating structural effect. | Exploratory inflammatory/hematologic toxicity signals in the present study; interpret according to the final harmonized results table and discussion. | Indirect concordance | Exploratory |

Concordance was classified according to whether the observed association matched a previously described drug–gene–phenotype relationship directly, was biologically plausible but indirect, or should be considered exploratory/hypothesis-generating. Interpretation strength reflects the extent to which the available literature supports the observed phenotype for the corresponding locus.
